# Comparison of golden-angle radial sparse parallel (GRASP) and conventional cartesian sampling in 3D dynamic contrast-enhanced mri for bladder cancer: a preliminary study

**DOI:** 10.1007/s11604-024-01637-w

**Published:** 2024-08-01

**Authors:** Yoshiko Ueno, Keitaro Sofue, Tsutomu Tamada, Mitsuru Takeuchi, Naoya Ebisu, Kentaro Nishiuchi, Takuto Hara, Toshiki Hyodo, Hideaki Miyake, Takamichi Murakami

**Affiliations:** 1 Department of Radiology, Kobe University Graduate School of Medicine, 7-5-2, Kusunoki-Cho, Chuo-Ku, Kobe, Hyogo 650-0017 Japan; 2 Department of Radiology, Kawasaki Medical School, 7577 Matsushima, Kurashiki, Okayama 701-0192 Japan; 3 Radiolonet Tokai, Chome-86 Asaokacho, Chikusa Ward, Nagoya, Aichi 464-0811 Japan; 4 Department of Urology, Kobe University Graduate School of Medicine, 7-5-2, Kusunoki-Cho, Chuo-Ku, Kobe, Hyogo 650-0017 Japan; 5 Department of Pathology, Kobe University Graduate School of Medicine, 7-5-2, Kusunoki-Cho, Chuo-Ku, Kobe, Hyogo 650-0017 Japan

**Keywords:** Golden, Angle radial sparse parallel (GRASP), Dynamic contrast enhanced magnetic resonance imaging (DCE MRI), Bladder cancer, Vesical imaging reporting and data system (VIRADS)

## Abstract

**Purpose:**

To compare the image quality, inter-reader agreement, and diagnostic capability for muscle-invasive bladder cancer (MIBC) of the reconstructed images in sections orthogonal to the bladder tumor obtained by 3D Dynamic contrast-enhanced (DCE)-MRI using the Golden-angle Radial Sparse Parallel (GRASP) technique with the images directly captured using the Cartesian sampling.

**Materials and methods:**

This study involved 68 initial cases of bladder cancer examined with DCE-MRI (GRASP: n = 34, Cartesian: n = 34) at 3 Tesla. Four radiologists conducted qualitative evaluations (overall image quality, absence of motion artifact, absence of streak artifact, and tumor conspicuity) using a five-point Likert scale (5 = Excellent/None) and quantitative signal-to-noise ratio (SNR) and contrast-to-noise ratio (CNR) measurements. The areas under the receiver-operating characteristic curves (AUCs) for the Vesical Imaging-Reporting and Data System (VI-RADS) DCE score for MIBC assessment were calculated. Inter-reader agreement was also assessed.

**Results:**

GRASP notably enhanced overall image quality (pooled score: GRASP 4 vs. Cartesian 3, P < 0.0001), tumor conspicuity (5 vs. 3, P < 0.05), SNR (Median 38.2 vs. 19.0, P < 0.0001), and CNR (7.9 vs. 6.0, P = 0.005), with fewer motion artifacts (5 vs. 3, P < 0.0001) and minor streak artifacts (5 vs. 5, P > 0.05). Although no significant differences were observed, the GRASP group tended to have higher AUCs for MIBC (pooled AUCs: 0.92 vs. 0.88) and showed a trend toward higher inter-reader agreement (pooled kappa-value: 0.70 vs. 0.63) compared to the Cartesian group.

**Conclusions:**

Using the GRASP for 3D DCE-MRI, the reconstructed images in sections orthogonal to the bladder tumor achieved higher image quality and improve the clinical work flow, compared to the images directly captured using the Cartesian. GRASP tended to have higher diagnostic ability for MIBC and showed a trend toward higher inter-reader agreement compared to the Cartesian.

## Introduction

For optimal treatment strategies in bladder cancer, an accurate assessment of local staging is crucial [1, 2]. MRI serves as a valuable tool for determining the necessary extent and depth of resection in transurethral resection of bladder tumors (TURBT). [3–8]. Previous study has indicated that oblique sections intersecting both the tumor and muscle layer are beneficial for assessing muscle-invasive bladder cancer (MIBC) [24]. However, bladder cancer is often multifocal, making it challenging to image the optimal orthogonal sections for each tumor.

The Golden-angle RAdial Sparse Parallel (GRASP) sequence is a recently introduced imaging technique that combines Three-dimentional (3D) radial sampling, parallel imaging, and compressed sensing reconstruction for dynamic contrast-enhanced (DCE) MRI [9–11]. Reducing the number of spokes to improve temporal resolution in radial sampling usually results in streak artifacts. Compressed sensing, through iterative reconstruction processing, can remove these streak artifacts independent of data volume [12–14]. In the GRASP method, radial sampling is performed at a special angle known as the golden angle, approximately 111.25 degrees. This approach minimizes data bias within k-space, enabling the acquisition of high temporal resolution images without compromising image quality. GRASP is not only resistant to motion artifacts but also capable of reconstructing images at arbitrary phases from the acquired data. Excellent GRASP-derived images have been demonstrated with fewer motion and pulsation artifacts in prior liver, prostate, breast, and brain studies [15–18].

We hypothesized that the GRASP technique could facilitate the implementation of 3D DCE-MRI for assessing muscle invasion in bladder cancer by enabling post-processing reconstruction of arbitrary orthogonal sections. To ensure the novelty of the research content based on this hypothesis, we conducted a comprehensive literature search using PubMed, Embase, and Web of Science databases with the keywords ‘GRASP’, ‘bladder cancer’, ‘MRI’, and ‘orthogonal reconstruction’ (and their variations) for articles published up to January 2024. No studies were found that specifically examined the use of GRASP or similar techniques for orthogonal reconstruction in bladder cancer imaging. Therefore, the aim of this study was to evaluate the utility of 3D DCE-MRI obtained using the GRASP method by comparing image quality, diagnostic ability for MIBC, and inter-reader agreement with those of the conventional Cartesian sampling method.

## Materials and methods

### Compliance statement

This study was conducted with the approval of the Ethics Committee at Kobe University Hospital. As it was a retrospective observational study, the requirement to obtain informed consent from patients was waived. This study was carried out in accordance with the principles of the Declaration of Helsinki, which guides medical research involving human subjects, ensuring ethical standards are met even in the absence of informed consent.

### Subjects

From May 2022 to January 2024, patients clinically suspected of primary bladder cancer underwent multiparametric MRI before TURBT at a 3 T MRI facility at Kobe University Hospital. This included T2-weighted imaging (T2WI), Diffusion-weighed imaging (DWI), and DCE imaging using the GRASP technique. A study coordinator, Y.U. (a radiologist with 13 years of experience in urogenital MRI), reviewed the medical records and pathological reports of these patients. The inclusion criteria for image quality assessment were defined as follows: (1) cases with a complete sequence of DCE-MRI using GRASP, (2) cases in which TURBT had been performed within three months post-MRI, and (3) tumors identifiable on MRI. Initially, 52 patients met these criteria. Subsequently, cases were excluded for the precise assessment of diagnostic capability in MIBC if they involved: (1) cases where repeat TURBT was not conducted appropriately according to EAU Guidelines [1, 2, 19] (n = 10), (2) cases lacking pathological specimens necessary for the assessment of muscle invasion in bladder cancer (n = 7), and (3) cases treated with chemotherapy prior to TURBT (n = 1). As a result, 34 cases were deemed eligible for the GRASP group. Among these cases, there were 15 instances of MIBC.

**Fig. 1:**
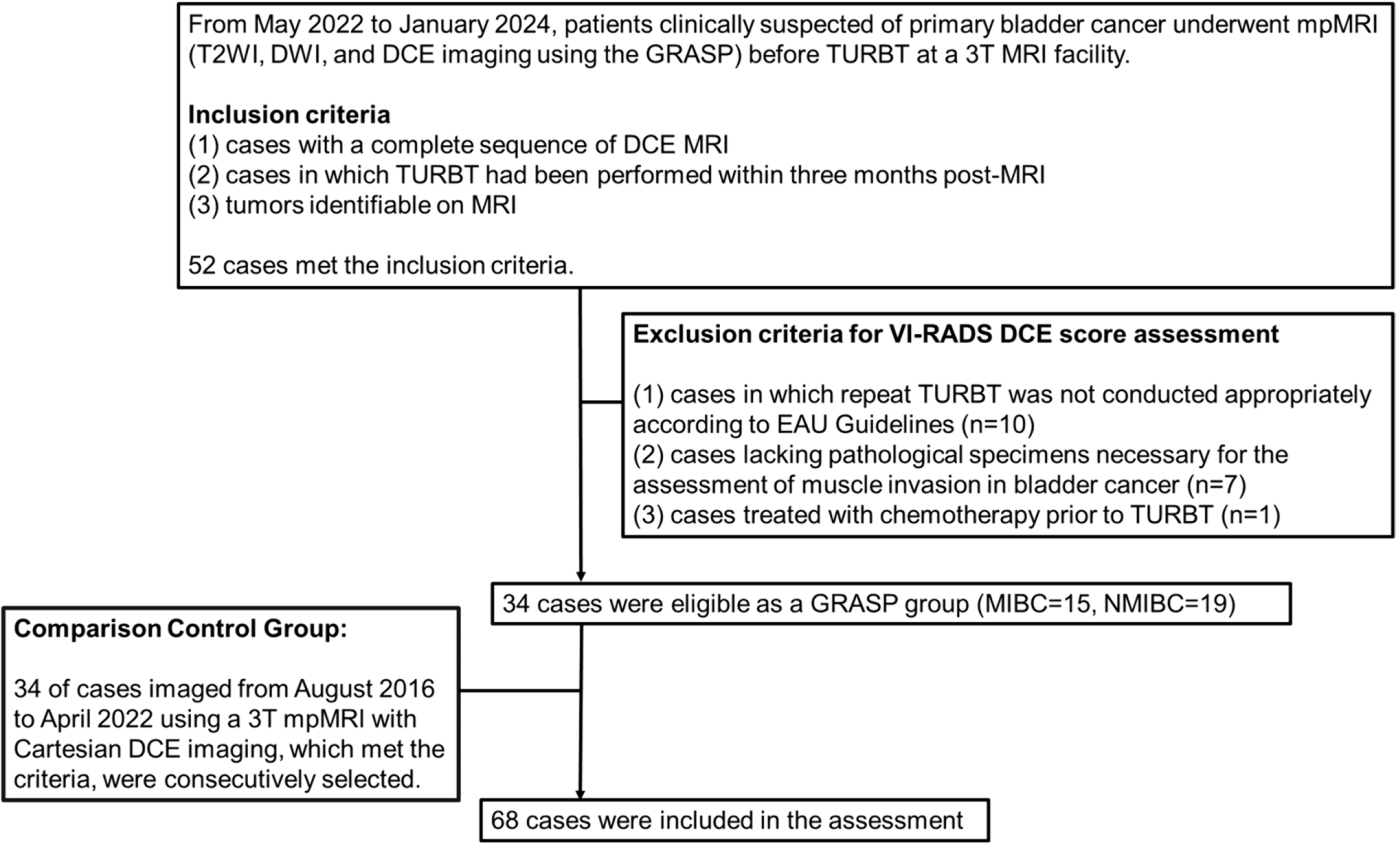
Flowchart of patients’ inclusion and exclusion. A total of 68 cases were utilized for the assessment

To compare the GRASP method with the Cartesian method, a retrospective analysis was conducted from January 2021 to August 2016, examining cases clinically suspected of initial bladder cancer, imaged using a 3 T MRI with T2WI, DWI, and DCE imaging using the Cartesian sampling method. Cases meeting the aforementioned inclusion and exclusion criteria were selected and then consecutively extracted to ensure sufficient numbers for a comparative control. Ultimately, 68 cases were examined in this study (GRASP: n = 34, Cartesian: n = 34). The flowchart of patient inclusion and exclusion is shown in Fig. 1.

### MRI technique

MRI examinations were performed using a 3 T machine equipped with a body array coil. Detailed MRI parameters are presented in Table 1. The GRASP technique was imaged using an MRI (MAGNETOM Vida 3 T, Siemens Healthcare, Germany), while the Cartesian method utilized MRIs (Achieva 3 T or Ingenia 3 T CX Quasar Dual, Philips Medical Systems, The Netherlands). T2WI was utilized for anatomical assessment. DWI was not employed in this analysis. DCE-MRI utilizing the GRASP technique was captured in the axial plane relative to the body axis and reconstructed in a plane orthogonal to the maximum dimension of the lesion. Regarding temporal resolution, the horizontal plane images were reconstructed in 10 s, while the cross-sectional images orthogonal to the tumor were reconstructed in 30 s. The reconstructed slice thickness was set to 1.6 mm. Cartesian DCE-MRI was acquired directly only in the plane orthogonal to the largest lesion. DCE imaging of the pelvis was performed post-administration of 0.1 mmol/kg body weight of gadolinium chelate (gadobutrol, Gadovist, Bayer). Prior to all MRI examinations, 20 mg of hyoscine butylbromide (Buscopan, Boehringer Ingelheim) was administered intramuscularly to reduce bowel peristalsis.

**Table 1:** Parameter of MRI

Parameter	Sequence	Imaging direction	TR/TE (msec)	Matrix	NEX	FOV (cm)	Slice thickness (mm)	b-value (sec/mm^2^)	Temporal resolution (sec)	Pre-scan time (sec)	Imaging duration (sec)
T2WI	FSE	Axial, sagittal, coronal, oblique axial	2000–5380 / 80–100	320 × 320—336 × 336	1–4	20–25	4	–	–		
DWI	EPI	Axial	5000–8000 / 70–82	128 × 154	1–4	24–36	4	0, 1000	–		
DCE-T1WI (GRASP)	3D-GRE	Axial	3.1 / 4.4	288	1	26–35	0.8 (re) / 1.6 (aq)	–	10	60	180*
DCE-T1WI (Cartesian)	3D-GRE	Oblique axial	3.9–5.5 / 1.4–3.3	230 × 288–320 × 336	1	26–35	1.5 (re) / 3 (aq)	–	30	–	180

*TR* Repetition time, *TE* Echo time, *NEX* Number of excitations, *FOV* Field of view, *T2WI*  T2 weighted imaging, *FSE* Fast spin echo, *DWI* Diffusion weighted imaging, *EPI* Echo planar imaging, *DCE-T1WI* Dynamic contrast enhanced T1 weighted imaging, *GRASP* Golden-angle RAdial Sparse Parallel, *GRE* Gradient echo, *3D* Three dimensional, *re* reconstructed, *aq* acquired. *Excluding the pre-scan time

### Data analysis

#### Patient and tumor characteristics

To evaluate whether there were any clinical factors influencing the assessment between the GRASP and Cartesian groups, we compared patients’ sex, age, days from MRI to TURBT, tumor histology, multiplicity or singularity of tumors, T stage, and pathological grade. Additionally, the number of cases requiring real-time on-table monitoring by a radiologist to determine the optimal imaging plane for the tumor during imaging was also compared.

### Qualitative assessment

The images of Cartesian DCE-MRI, directly taken in sections orthogonal to the tumor, and the images of GRASP DCE-MRI, reconstructed in sections orthogonal to the tumor post-imaging, were subjectively evaluated by four radiologists (N.E., K.N., M.T., and T.T., designated as readers A, B, C, and D with 3, 3, 19, and 23 years of experience in urogenital MRI, respectively) using a five-point Likert scale. The evaluation criteria included overall image quality, absence of motion artifacts, absence of streak artifacts, and tumor conspicuity. Evaluation of overall image quality and tumor conspicuity was performed using a five-point Likert scale as follows: 1 = Nondiagnostic, 2 = Poor, 3 = Satisfactory, 4 = Good, 5 = Excellent. For the assessment of the absence of motion artifacts and absence of streak artifacts, the following scale was applied: 1 = Nondiagnostic, 2 = Suboptimal for diagnosis, 3 = Obvious quality degradation but diagnostic, 4 = Minor quality degradation, 5 = No degradation in quality. The evaluators were blinded to whether the images belonged to the GRASP or Cartesian group, and the images were assessed in a random order regardless of the group.

### Quantitative assessment

Two board-certified radiologists (Y.U. and K.S., with 13 and 19 years of experience in urogenital MRI, respectively) measured the signal-to-noise ratio (SNR) and the contrast-to-noise ratio (CNR) of the bladder and tumor in consensus. Measurements were taken during the phase of the dynamic MRI where the contrast between the tumor and muscle layer was most visually distinct. When multiple tumors were detected, the largest single tumor was analyzed. Regions of interest (ROIs) were manually drawn to profile the tumor on the layer with the largest tumor size, avoiding necrosis, artifacts, blood vessels, and tumor stalks. In the same layer, an ROI of the urine was delineated using unified circular sampling (voxel count = 300). Then, the mean signal amplitude of the tumor (SI_tumor_) and its standard deviation (σ_tumor_) were recorded, as well as those of the urine (SI_urine_) and (σ_urine_), for each patient. The relative SNR was calculated using Eq. (1), and the CNR using Eq. (2) [20].

1 $$SNR = \frac{{SI_{{{\text{tumor}}}} }}{{\sigma_{{{\text{urine}}}} }}$$

2 $$CNR = \frac{{\left| {SI_{{{\text{tumor}}}} - SI_{{{\text{urine}}}} } \right|}}{{\sqrt {\sigma_{{{\text{tumor}}}}^{2} + \sigma_{{{\text{urine}}}}^{2} } }}$$

### VI-RADS DCE scoring for assessment of MIBC

The Vesical Imaging-Reporting and Data System (VI-RADS) was utilized for MIBC assessment [8]. VI-RADS scores for DCE were assessed by the four radiologists (reader A, B, C, and D) on dynamic MRI images using the GRASP (n = 34) and the Cartesian sampling method (n = 34), based on the following definitions [8]: category 1: No early enhancement of the muscularis propria or less than 1 cm in size, category 2: no early enhancement of muscularis propria with early enhancement of inner layer, and greater than 1 cm in size, category 3: lack of category 2 findings but with no clear disruption of low SI muscularis propria, category 4: tumor early enhancement extends focally to muscularis propria but to extravesical fat tissue, category 5: tumor early enhancement extends to the entire bladder wall and to extravesical fat. In the GRASP group, both directly captured images and images reconstructed in planes orthogonal to the tumor were utilized.

### Statistical analysis

All statistical analyses were conducted using JMP software, version 13.0 (SAS Institute Inc., Cary, NC, USA). Differences between the GRASP and Cartesian groups were evaluated using the Mann–Whitney U test for nonparametric data. P-values less than 0.05 were considered statistically significant. Subsequently, the diagnostic abilities of the VI-RADS DCE scores for MIBC in each group were analyzed using ROC curves to determine the area under the curve (AUC) and the optimal cutoff values for sensitivity, specificity, and accuracy. Using the bootstrap method (1000 iterations), the difference in the AUC between groups was estimated, and the mean of these differences along with their 95% confidence intervals was computed. If the 95% confidence interval included zero, the difference in AUC between the two groups was concluded to be not statistically significant [21, 22].　Inter-reader agreement for VI-RADS DCE scores was assessed using linear weighted Kappa statistics, with results interpreted as follows: slight agreement (0–0.20), fair agreement (0.21–0.40), moderate agreement (0.41–0.60), substantial agreement (0.61–0.80), and excellent agreement (0.81–1.00) [23].

## Results

### Patient and tumor characteristics

**Fig. 2:**
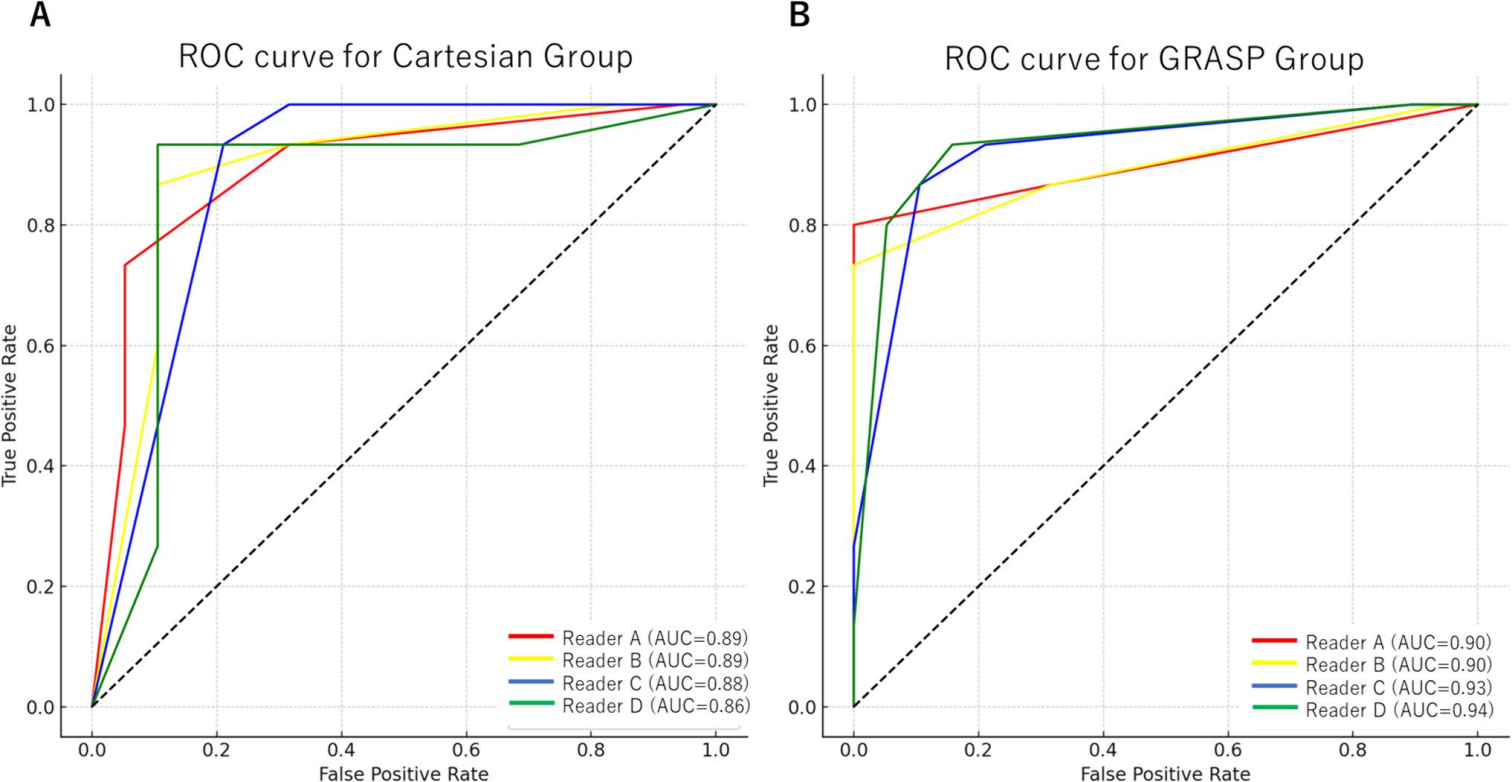
ROC curves demonstrating the diagnostic capability of muscle-invasive bladder cancer using the VI-RADS DCE score. The results obtained using the Cartesian technique, with Area Under the Curve (AUC) values of 0.89 for reader (**A**), 0.89 for reader (**B**), 0.88 for reader (**C**), and 0.86 for reader (**D**). The results using the GRASP technique, with AUC values of 0.90 for reader (**A**), 0.90 for reader (**B**), 0.93 for reader (**C**), and 0.94 for reader (**D**)

The characteristics of the patients and tumors are shown in Table 2. No significant differences were observed between the groups regarding patient gender, age, days from MRI examination to TURBT, histological type of the tumor, whether the tumor is solitary or multiple, number of cases with muscle invasion, grade, or maximum tumor diameter (P > 0.05). The number of cases requiring real-time on-table monitoring by a radiologist during imaging was significantly higher in the Cartesian group compared to the GRASP group (Cartesian: 11 vs. GRASP: 0, P < 0.0001) (Fig. 2).

**Table 2:** Patient and tumor characteristics

	GRASP	Cartesian	P-value
Patient characteristics (n = 68)			
Sex			1.00
Male	25	27	
Female	9	7	
Age (y)*	72.3 [56,87] (10.2)	75.7 [58,85] (12.0)	0.15
Interval between MRI and subsequent TURBT (day)*	21.0 [3,66] (13.5)	23.0 [4,64] (17.0)	0.93
Cases requiring on-table monitoring by a radiologist	0	11	< 0.0001
Tumor characteristics			
Histology			1.00
Urothelial carcinoma	34	34	
Whether the tumor is solitary or multiple			0.12
Solitary	23	10	
Multiple	11	24	
Highest T stage			0.70
Ta	10	9	
T1	9	10	
T2 or greater	15	15	
Histologic grade			0.70
Low	31	28	
High	3	6	
Tumor maximum diameter (mm)*	23.0 [6,65] (28.5)	25.0 [4,96] (21.0)	0.53

*GRASP* golden-angle radial sparse parallel, *TURBT* Transurethral resection of bladder tumor

*indicates Median [range] (interquartile). Other data in cells indicate the number

### Qualitative assessment

**Fig. 3:**
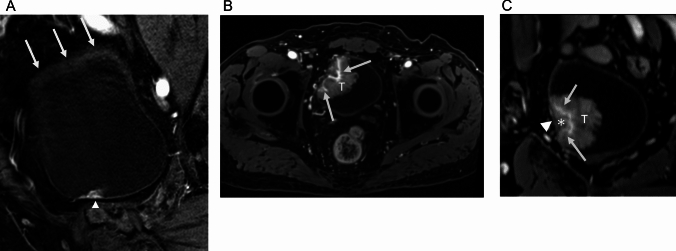
Cases of non-muscle invasive bladder cancer. **A** Dynamic Contrast-Enhanced MRI (DCE-MRI) acquisition using Cartesian method at 60 s post-contrast injection. The DCE-MRI displays significant intestinal motion artifacts on the bladder’s superior wall (arrows). The tumor located on the inferior wall of the bladder allows for clear evaluation. Preservation of inner layer enhancement at the tumor base (arrowhead) supports the diagnosis of non-muscle invasive bladder cancer. Pathological findings from transurethral resection of the bladder tumor (TURBT) confirmed a T1 high-grade cancer. **B** DCE-MRI acquisition using GRASP method, axial section at 60 s post-contrast injection. The tumor is observed on the right wall of the bladder (labeled as T), with retention of inner layer enhancement indicated (arrows). **C** Reconstructed cross-sectional image where tumor (labeled as T) intersects with muscle layer (arrowhead). This reconstruction facilitates a detailed assessment of the inner layer enhancement (arrows) and the stalk (marked by *). Pathological examination from TURBT identified the tumor as Ta low grade

**Fig. 4:**
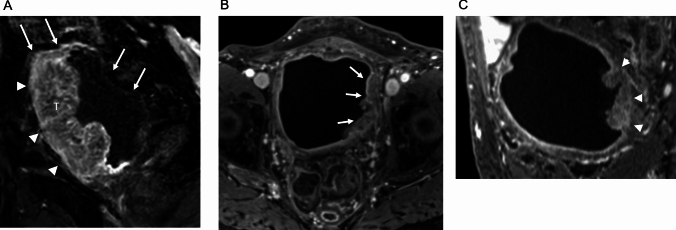
Cases of muscle-invasive bladder cancer. **A** Dynamic contrast-enhanced MRI (DCE-MRI) acquired using Cartesian technique at 90 s post-contrast injection. This DCE-MRI shows significant intestinal motion artifacts on both the superior and posterior walls of the bladder (arrows). A widespread tumor (labeled as T) is visible on the anterior wall of the bladder. The tumor projects beyond the bladder wall, suggesting infiltration into the adipose tissue (arrowheads). The pathological findings from transurethral resection of the bladder tumor (TURBT) indicated a stage of T2 or higher. **B** DCE-MRI acquired using GRASP technique, axial section at 90 s post-contrast injection. The tumor is located on the left wall of the bladder (arrows) and is broad-based. **C** Reconstructed cross-sectional image at the intersection of tumor and muscle layer. This image clearly shows disruption of the inner layer enhancement at the base of the tumor (arrowheads). Pathological examination from TURBT confirmed a stage of T2 or higher

The results are presented in Table 3. In the qualitative assessment of image quality, all readers indicated significantly higher scores for overall image quality (pooled score: GRASP 4 vs Cartesian 3 P < 0.0001), absence of motion artifacts (pooled score: GRASP 5 vs Cartesian 3, P < 0.0001), and tumor conspicuity (pooled score: GRASP 5 vs Cartesian 3, P < 0.05 in the GRASP group. Regarding the absence of streak artifacts, no significant difference was observed between the GRASP and the Cartesian method across all readers. (pooled score: GRASP 5 vs Cartesian 5, P > 0.05). The cases are presented in Figs. 3 and 4.

**Table 3:** Comparison of qualitative assessment of image quality

	GRASP Median [range] (interquartile)	Cartesian Median [range] (interquartile)	P-value
Overall image quality			
Reader A	4 [4, 5] (1)	3 [2, 5] (1)	< 0.0001
Reader B	4 [4, 5] (0)	3 [2, 4] (1)	< 0.0001
Reader C	4 [4, 5] (1)	3 [3, 4] (1)	< 0.0001
Reader D	5 [3, 5] (0)	4 [2, 5] (1.25)	< 0.0001
Absence of streak artifact			
Reader A	5 [3, 5] (1)	5 [5] (0)	0.17
Reader B	5 [4, 5] (1)	5 [3, 5] (0)	0.17
Reader C	5 [3, 5] (1)	5 [4, 5] (0)	0.33
Reader D	5 [4, 5] (0)	5 [4, 5] (0)	1.00
Absence of motion artifact			
Reader A	5 [4, 5] (0)	3 [2, 5] (1)	< 0.0001
Reader B	5 [4, 5] (0)	3 [2, 5] (2)	< 0.0001
Reader C	5 [3, 5] (0)	3 [2, 5] (0)	< 0.0001
Reader D	5 [4, 5] (0)	3 [2, 5] (1)	< 0.0001
Tumor conspicuity			
Reader A	5 [3, 5] (0)	3 [2, 5] (1)	< 0.0001
Reader B	5 [3, 5] (0)	3 [3, 5] (1)	< 0.0001
Reader C	5 [3, 5] (0)	3 [2, 5] (1)	< 0.0001
Reader D	5 [3, 5] (0)	3 [2, 5] (1)	< 0.0001

*GRASP* Golden-angle radial sparse parallel

### Quantitative assessment

The results are displayed in Table 4. Both SNR and CNR were significantly higher in the GRASP group (SNR: GRASP 38.2 vs. Cartesian 19.0 (Median), P < 0.0001, CNR: GRASP 7.9 vs. Cartesian 6.0, P = 0.005).

**Table 4:** Comparison of quantitative assessment of image quality

Parameter	GRASP Median [range] (interquartile)	Cartesian Median [range] (interquartile)	P-Value
SNR	38.2 [12.1, 73.3] (14.1)	19.0 [6.3, 45.4] (8.5)	< 0.0001
CNR	7.9 [2.0,10.6] (2.6)	6.0 [1.6, 9.2] (2.6)	0.005

*SNR* Signal to noise ratio, *CNR* Contrast to noise ratio

### VI-RADS DCE scoring for assessment of MIBC

Among the all radiologists, the AUC was higher for the GRASP group compared to the Cartesian group (GRASP vs. Cartesian: Reader A, 0.90 vs. 0.89, Reader B, 0.90 vs. 0.89, Reader C, 0.92 vs. 0.88, Reader D, 0.93 vs. 0.86, respectively). However, the 95% confidence intervals for the AUC of the GRASP and Cartesian groups overlapped for all evaluators, and no significant difference was considered to exist. The results are shown in Table 5 and Fig. 2.

**Table 5:** Comparison of the diagnostic capability of the VI-RADS DCE score

	AUC [95% CI]	Mean difference [95% CI]	Sensitivity (number)	Specificity (number)	Optimal Threshold
GRASP					
Reader A	0.90 [0.74, 1]	0.05 [– 0.13, 0.24]	0.8 (12/15)	1 (19/19)	4
Reader B	0.90 [0.74,1]	– 0.007 [– 0.17, 0.15]	0.73 (11/15)	1 (19/19)	4
Reader C	0.93 [0.75,1]	– 0.035 [– 0.18, 0.10]	0.87 (13/15)	0.89 (17/19)	4
Reader D	0.94 [0.85,1]	– 0.075 [– 0.27, 0.09]	0.93 (14/15)	0.84 (16/19)	3
Cartesian					
Reader A	0.89 [0.73,1]	–	0.73 (11/15)	0.95 (18/19)	4
Reader B	0.89 [0.74,1]	–	0.87 (13/15)	0.89 (17/19)	4
Reader C	0.88 [0.70,1]	–	0.93 (14/15)	0.79 (15/19)	4
Reader D	0.86 [0.66,1]	–	0.93 (14/15)	0.89 (17/19)	4

*VI-RADS*  Vesical imaging-reporting and data system, *DCE* Dynamic contrast enhanced, *GRASP* Golden-angle RAdial Sparse Parallel, *AUC* Area under the curve, *CI* confidential interval

### Inter-reader agreement

The results are shown in Table 6. The inter-reader agreement rates tended to be higher in the GRASP group compared to the Cartesian group across all combinations of readers. Notably, the agreement rate among readers with extensive reading experience (reader C and D) was excellent in the GRASP group (kappa value 0.89).

**Table 6:** Inter-reader agreement

Pair	GRASP		Cartesian	
	Weighted kappa score	95% CI	Weighted kappa score	95% CI
Reader A vs B	0.78	[0.63, 0.89]	0.63	[0.42, 0.79]
Reader A vs C	0.62	[0.41, 0.77]	0.60	[0.41, 0.75]
Reader A vs D	0.63	[0.45, 0.77]	0.62	[0.45, 0.74]
Reader B vs C	0.64	[0.46, 0.77]	0.53	[0.28, 0.71]
Reader B vs D	0.67	[0.50, 0.78]	0.66	[0.49, 0.78]
Reader C vs D	0.89	[0.77, 0.97]	0.74	[0.54, 0.87]

*GRASP* Golden-angle RAdial Sparse Parallel, *CI* confidential interval. Reader

## Discussion

Our multi-reader study results suggest that GRASP has the potential to enhance the image quality of bladder MRI. Additionally, the GRASP method enabled the acquisition of 3D DCE-MRI with a slice thickness thin enough to obtain arbitrary reconstructed sections. GRASP employs a radial scanning technique that collects data in a rotating fashion, thereby dispersing motion artifacts more effectively compared to the Cartesian method. Each acquisition with GRASP captures signals from the center of k-space, leading to averaging that enhances image contrast. In Addition, the reconstruction of weighted data from GRASP can be expected to yield a higher SNR. Our results were consistent with these theoretical advantages, showing significant improvements in overall image quality, tumor conspicuity, SNR, and CNR with GRASP, despite the presence of streak artifacts which were clinically negligible. Furthermore, in the GRASP group, there were no cases that required real-time on-table monitoring by a radiologist to determine the optimal imaging plane for the tumor. Particularly in cases with multiple tumors, obtaining the optimal oblique sections using Cartesian sampling may require radiologist supervision during the examination. When using the GRASP technique, imaging is performed in the horizontal plane relative to the patient’s body axis, and optimal sections for each tumor can be reconstructed afterward. This approach has the potential to improve the radiology workflow and enhance efficiency.

The diagnostic capability of MIBC using the VI-RADS DCE score demonstrated high AUC values, with the GRASP cohort achieving 0.90–0.93 and the Cartesian cohort achieving 0.86–0.89. In this investigation, no scores were assigned for DWI, and hence the diagnostic pathway does not strictly conform to the VI-RADS criteria. However, the outcomes displayed are similar to the diagnostic efficacy of VI-RADS as previously reported in the literature [25–27]. Although all radiologists exhibited slightly higher AUC values in the GRASP group compared to the Cartesian group, no significant differences were observed between these cohorts. The lack of significant difference in diagnostic performance between the GRASP group and the Cartesian group can be attributed to the fact that the evaluation of diagnostic performance was based solely on the largest lesion, even in cases with multiple bladder cancers. Furthermore, our post hoc review suggested that there were relatively fewer instances in the Cartesian group where artifacts from intestinal peristalsis overlapped with the tumors. In both groups, the difference in diagnostic performance based on the years of reading experience was not evident. Previous studies have also reported that the diagnostic performance of VI-RADS is not significantly influenced by the years of reading experience [28], and our results are consistent with these findings. We assume that this can be attributed to the standardized nature of the VI-RADS reading method. The inter-reader agreement tended to be higher in the GRASP group compared to the Cartesian group, which can be attributed to the superior image quality achieved with the GRASP method.

This study has a few limitations, including its single-center, retrospective comparative nature with different patient groups, and relatively small cohort size due to stringent patient selection criteria. Additionally, no comparisons between DCE scores and DWI scores in VI-RADS were conducted. While VI-RADS considers DWI as the dominant sequence, single-shot EPI DWI, commonly used, is susceptible to distortion and artifacts from bowel peristalsis. DCE-MRI can serve as a powerful diagnostic tool in bladder imaging when the quality of DWI is suboptimal. Since this study is an initial investigation of GRASP in clinical settings for bladder MRI, future studies that address and complement the aforementioned points are desirable.

In conclusion, the GRASP technique has the potential to enhance the image quality of bladder DCE MRI. Although no significant differences were observed, the GRASP method tended to have higher diagnostic ability for MIBC and showed a trend toward higher inter-reader agreement compared to the traditional Cartesian method. The GRASP’s ability to reconstruct optimal sections tailored to each case indicates its potential to improve clinical workflow.
